# Supplementary material for: Patterns of Intron Gain and Loss in Fungi
Source: PLoS Biol. 2004 Nov 30;2(12):e422. doi: 10.1371/journal.pbio.0020422 (PMC532390; doi:10.1371/journal.pbio.0020422)
Supplement: Table S1 — Also available at http://genes.mit.edu/NielsenEtAl/. (4.3 MB ZIP). [file pbio.0020422.st001.zip › NielsenEtAl/html/860.html]

AN2295.1.NCU01227.1.MG05480.1.FG02030.1


```
 CLUSTAL W (1.82) Multiple Sequence Alignments - Introns Inserted


Sequence 1: NCU01227.1	333 aa
Sequence 2: MG05480.1	333 aa
Sequence 3: FG02030.1	329 aa
Sequence 4: AN2295.1	329 aa
Alignment Length: 333 aa
Number Identitical Residues: 246 aa
Alignment Score (without introns) 10150


MG05480.1 	MQAISRQLRPSGAILRQISTRTYSSSSSPYAATINNLRINSDTKVLFQGFTGKQGT2FHA
NCU01227.1	MQAIARQTRPAGKLVQQLSKRAYSSNSSPYAATINNLKINGDTKVLFQGFTGKQGT2FHA
FG02030.1 	MQSITRATRAS--ALKNVGRRAYSS--SQYAKTINNLRINSDTKVIYQGFTGKQGT2FHA
AN2295.1  	MQAFRRNAVSALRNAAATQRRAYTP--GSYAETVNNLRINADTKVLFQGFTGKQGT2FHA
          	**:: *   .:         *:*:.  . ** *:***:**.****::********* ***

MG05480.1 	QQAIDYG1TQVVGGTNPKKAGQTHLDLPVFANVADGVKQTGANATAIFVP2PPLAAAGIE
NCU01227.1	QQAIEYG1TNVVGGTNPKKAGQTHLDRPVFSNVSEAVKETGATATAIFVP2PPLAAAGIE
FG02030.1 	EQAIEYG1TKVVGGTNPKKAGQTHLGLPVFKNVSEAVKATGATATGLFVP2PPLAAAGIE
AN2295.1  	EQAIAYG1TKVVGGTNPKKAGSTHLDLPVFANVSEAVKETGATASAIFVP2PPLAAKGIE
          	:*** ** *:***********.***. *** **::.** ***.*:.:*** ***** ***

MG05480.1 	EAVAAEVPLVVC~ITEGIPQHD1MVRITSILKSQNKTRLVGPNCPGIIAP~GQCKIGIMP
NCU01227.1	EALQAEIPLAVC~ITEGIPQHD1MVRITSMLKTQSKTRLVGPNCPGIIAP~GQCKIGIMP
FG02030.1 	EALEAEIPLVVC~ITEGIPQHD1MVRITDMLKTQDKTRLVGPNCPGIIAP~EQCKIGIMP
AN2295.1  	EAIEAEVPLVVC2ITEGIPQHD1MVRITDILKTQNKTRLVGPNCPGIIAP0GQCKIGIMP
          	**: **:**.** ********* *****.:**:*.***************  ********

MG05480.1 	GFIHKRGRIGIVSRSGTLTYEAVNQTTQAGLGQSLVVGIGGDPFSGTNFIDCLKVFLQDG
NCU01227.1	GFIHKRGRIGIVSRSGTLTYEAVNQTTQAGLGQSLVVGIGGDPFSGTNFIDCLKVFLEDG
FG02030.1 	GFIHKRGRIGIVSRSGTLTYEAVNQTTQAGLGQSLVVGIGGDPFSGTNFIDCLKVFTEDP
AN2295.1  	GFIHKRGRVGIVSRSGTLTYEAVNQTTQAGLGQSLVVGIGGDPFSGTNFIDCLRIFLEDE
          	********:********************************************::* :* 

MG05480.1 	ETDGIIMIGEIGGSAEEEAADFLRANNTVNGGKPVVSFIAGISAPAGRRMGHAGAIVSGG
NCU01227.1	DTDGIIMIGEIGGSAEEEAADFLKEYNTKNGGKPVVSFIAGISAPPGRRMGHAGAIVAGG
FG02030.1 	NTDGIIMIGEIGGSAEEDAAEFLKQANTVNGGKPVVSFIAGISAPPGRRMGHAGAIVSGG
AN2295.1  	ETDGIIMIGEIGGSAEEDAAEFFKANNKHN--KPAVGFIAGISAPPGRRMGHAGAIVSGG
          	:****************:**:*::  *. *  **.*.********.***********:**

MG05480.1 	KGGADSKIKALEAAGVIVEPSPAGLGKALRDEFVRRDLL
NCU01227.1	KGGADSKIKALEAAGVVVERSPASLGKALRDEFVRRDLL
FG02030.1 	KGGADTKIKALEDAGVIVERSPAGLGKALYNEFVRRDLL
AN2295.1  	KGGADSKISALESAGVVVERSPASLGKSLLAEFVKRDLV
          	*****:**.*** ***:** ***.***:*  ***:***:
```
